# Book Reviews

**Published:** 1897-10

**Authors:** 


### BOOK REVIEWS.

Eye-Strain in Health and Disease. With Special Reference to the Amelioration or Cure of Chronic Nervous Derangements without the Aid of Drugs. By Ambrose L. Ranney, A.M., M.D., author of "Lectures on Nervous Diseases," "The Applied Anatomy of the Nervous System," etc., etc. Illustrated with 38 wood-cuts. Extra cloth, beveled edges, $2 net. The F. A. Davis Co., Publishers, 1914–1916 Cherry Street, Philadelphia.

This book is intended perhaps more for the general practitioner than for the specialist. It is an attempt to draw the attention of medical men to the relationship which exists, in the opinion of the author, between defects in refraction or ocular muscular balance, possibly unfelt by the patient, and many diseases of a serious nature affecting various departments of the physical economy. He does not simply expound a theory, but his words are the result of personal experience, and therefore worthy of careful attention. Every ophthalmologist knows how frequently headaches and neuralgia, and even dizziness and vomiting, are cured by attention to the eyes, and how intractable are many local ocular diseases until the refraction has been treated. But it has been a question often discussed whether eye-strain is capable of causing or accentuating other and more serious complaints. This author stands forth as a champion on the positive side, and brings forward a goodly array of cases from his own note-book which, so far as they go, appear amply to support his contentions. He explains the relationship indicated by the theory that the excess of nerve force used up by the ocular strain, diminishes the amount available for the functions of other parts of the body. Besides headache and neuralgia, the diseases which he specially considers as frequently curable by ocular treatment are chorea, sleeplessness, digestive disturbances, epilepsy, nervous prostration and insanity. Now, positive is of much greater moment than negative evidence, and Dr. Ranney, who appears to be a careful observer, has cured many most serious and apparently hopeless cases of these diseases by the means under discussion. That some others have not been so successful may be because they have employed less thorough and patient trials, for it is no easy matter for the uninitiated to know what to do or how to do it.

Doubtless in time the truth will be arrived at when a sufficient mass of material will have been accumulated from different sources, but we heartily indorse this author in his advice to parents to have the eyes of their children examined early as a preventive measure, though we are far from desiring every child with some refractive error, *even without symptoms*, to wear glasses or undergo tenotomy. We believe the day will come when preventive medicine will include an examination of most of the important organs of the body at certain intervals, just as at present a visit, at least annually, to the dentist is recognized as wisdom by the majority of people. At the same time that we admit the possibility of his entire contention, we think the author would have better forwarded it by erring on the side of safety, for when he makes such statements as that gout and phthisis are, even indirectly, at times, due to eye-strain, and that if all anomalies of refraction and muscular equilibrium had been thoroughly rectified in youth, "many of the hopeless sufferers from phthisis that we all have met would have escaped the disease," he probably alienates the sympathies of a large proportion of his readers, and diminishes the number of his adherents. The book is one which is certainly worth the attention of the practitioner, for in it he may obtain many hints of value.

A. W. S.

---

PRINCIPLES OF MEDICINE. By Chas. S. Mack, M.D., Professor of Materia Medica and Therapeutics in the Hahnemann Medical College, Chicago. W. T. Keener Company, 96 Washington street, Chicago. [Price $1.00.]

The author of this little book teaches that "as a system of curative medicine homeopathy is exclusive," that a "cure cannot be intelligently attempted excepting under guidance of *similia similibus curantur* as law"; though he is gracious enough to concede that there may be "many principles (concerning which *similia* says nothing) upon which one can base useful though not curative treatment." One would suppose that this wholesome and unerring *law* of homeopathy would comprehend in its ample operations all necessary therapeutic measures, but we are told that a "very large majority of us homeopaths do not repudiate practices which we think useful, although they are not instances of homeopathy." Again, although homeopathy is a *law* (and was of course *law* when it was "discovered" by Hahnemann), the author objects to the Organon as a text-book, and advises students to go slow on potencies. However, for those who like this sort of a book, it will doubtless be quite satisfactory.

